# Cyclin G2 in macrophages triggers CTL-mediated antitumor immunity and antiangiogenesis via interferon-gamma

**DOI:** 10.1186/s13046-022-02564-2

**Published:** 2022-12-24

**Authors:** Lu Liu, Jinlan Gao, Xuesha Xing, Meixi Jiang, Qi Liu, Shusen Wang, Yang Luo

**Affiliations:** The Research Center for Medical Genomics, Key Laboratory of Medical Cell Biology, Ministry of Education, School of Life Science, China Medical University, No.77 Puhe Road, Shenyang North New Area, Liaoning Province Shenyang, People’s Republic of China

**Keywords:** Cyclin G2, IFN-γ, Macrophage, Cancer, STAT1

## Abstract

**Background:**

IFN-γ is a key mediator of tumor immunity that can induce macrophage polarization to suppress tumor growth. Cyclin G2 functions as a tumor suppressor in various cancer cells; however, its role in macrophages remains unclear. This study aimed to investigate the role and underlying mechanisms of cyclin G2 in macrophages in vitro and in vivo.

**Methods:**

Mouse tumor models were used to determine the effect of cyclin G2 in macrophages on tumor growth in vivo following IFN-γ treatment. Immunohistochemistry staining, immunofluorescence staining and flow cytometry were used to evaluate the number of cytotoxic T lymphocytes (CTLs) and blood vessels in the mouse tumors. Moreover, the biological roles of cyclin G2 in macrophages with regard to CTL chemotaxis, cytotoxic function, and vascular endothelial cell tube formation were assessed using in vitro functional experiments. Immunoprecipitation (IP), real-time PCR, and enzyme-linked immunosorbent assays (ELISAs) were conducted to investigate the underlying mechanisms by which cyclin G2 regulates CTLs and vascular endothelial cells.

**Results:**

We found that cyclin G2 expression was upregulated in macrophages after IFN-γ treatment. Upregulated cyclin G2 inhibited lung and colon cancer growth by increasing the secretion of its downstream effector CXCL9, which promoted CTL chemotaxis and suppressed vascular endothelial cell tube formation. Moreover, cyclin G2 increased CXCL9 mRNA levels by promoting STAT1 nuclear translocation. In addition, cyclin G2 promoted the activation of the STAT1 signaling pathway, which was dependent on PP2Ac.

**Conclusions:**

Cyclin G2 is upregulated by IFN-γ in macrophages, promotes the secretion of CXCL9 to increase CTL chemotaxis and inhibit angiogenesis to suppress tumor growth. Our findings suggest that targeting cyclin G2 could benefit future immunotherapy.

**Supplementary Information:**

The online version contains supplementary material available at 10.1186/s13046-022-02564-2.

## Background

The dynamic interactions between tumor cells and the immune and endothelial cells in the tumor microenvironment are crucial for regulating the occurrence and development of tumors. Macrophages are one of the main cell populations of the tumor microenvironment [1], and the immunosuppressive tumor microenvironment that they induce is a key cause of treatment failure. Therefore, re-educating macrophages to reprogram tumor microenvironment is expected to become an effective strategy against cancer. Immunotherapy targeting tumor immune system reprogramming has been extensively studied in recent years [2], and interferon-γ (IFN-γ) is the preferred cytokine for stimulating antitumor immunity [3]. The US Food and Drug Administration (FDA) has approved IFN-γ for treating chronic granuloma and osteoporosis [4, 5]. In tumor immunity, IFN-γ-activated M1-like macrophages can enhance the antitumor effect of cytotoxic T lymphocytes (CTLs) [6], and macrophages activated by IFN-γ can inhibit tumor angiogenesis by regulating cytokine secretion [7]. However, mechanisms of IFN-γ activated macrophages on anti-tumor remains not completely clear.

CXCL9 is an effector molecule expressed by IFN-γ-induced macrophages [8] and a key factor in CTL recruitment into tumors [9]. CXCL9 also effectively inhibits angiogenesis [10, 11]. Signal transducer and activator of transcription 1 (STAT1) is the central mediator of IFN-γ-induced gene expression. The JAK-STAT1 pathway is activated by IFN-γ, resulting in the phosphorylation of tyrosine 701 of STAT1 in macrophages. This phosphorylation causes STAT1 nuclear translocation, where it regulates the transcription of its downstream genes, such as *CXCL9* [12]. Protein Phosphatase 2 Catalytic Subunit Alpha (PP2Ac) inhibits STAT1 entry into the nucleus [13–15].

Our previous study showed that cyclin G2 inhibits tumor progression in gastric cancer, oral squamous cell carcinoma, and glioma [16–18]. In the present study, we investigated how cyclin G2 in macrophages could alter the immunosuppressive tumor microenvironment during the IFN-γ reprogramming of macrophages. Our findings define a new function for cyclin G2 in macrophages — regulating CTLs and tumor angiogenesis. The novel targets of macrophage-based tumor therapy provide more options for tumor immunotherapy. Using a series of in vivo and in vitro experiments, we elucidated the molecular mechanisms underlying cyclin G2-mediated regulation of CXCL9 in macrophages after IFN-γ treatment. This further illuminate the mechanisms of IFN-γ regulating macrophages in tumor microenvironment. Our elucidation of the antitumor function and mechanisms of cyclin G2 in macrophages provides a new theoretical basis for the development of tumor therapy. These findings may help to promote tumor immunotherapy against macrophages, especially in the context of IFN-γ.

## Methods

### Cell culture

The human myeloid leukemia mononuclear (THP-1) and embryonic kidney (HEK-293) cell lines and mouse lung cancer (LLC) and colon cancer (MC38) cell lines were purchased from the Chinese Academy of Science Cell Bank (Shanghai, China). Human umbilical vein endothelial cells (HUVECs) and the mouse lymphatic endothelial cell line (SVEC4–10) were obtained from Procell Life Science & Technology Co., Ltd. HUVECs and HEK-293 cells were cultured in DMEM (Gibco, Carlsbad, CA, USA). LLC and SVEC4–10 cells were maintained in RPMI 1640 (Gibco, Grand Island, NY, USA). THP-1 cells were cultured in RPMI 1640 (Gibco, Grand Island, NY, USA) supplemented with 0.05 mM β-mercaptoethanol (Sigma–Aldrich). All culture media were supplemented with 10% fetal bovine serum (FBS, Biological Industries, Israel) and 1% penicillin/streptomycin (Gibco). All cells were cultured in a humidified 37 °C incubator with 5% CO_2_ [19].

### Generation of stable cell lines

The PFUGW-3FLAG-EGFP-Vector (Vector), PFUGW-3FLAG-EGFP-cyclin G2 (Flag-cyclin G2), PFUGW-EGFP-Nonsense (Nonsense), and PFUGW-EGFP-shcyclin G2 (shcyclin G2#1 and shcyclin G2#2) plasmids were purchased from GeneChem Co., Ltd. (Shanghai, China) and used to package lentiviruses for infecting THP-1 cells. Transfected THP-1 cells were selected with 3 μg/mL puromycin (Sigma–Aldrich, Santa Clara, CA, USA) to generate stable cell lines [16, 18]. Small interfering RNA (siRNA) for the knockdown of PP2Ac (siPP2Ac) and scrambled nontargeting siRNA (siNC) were purchased from Genepharma Co., Ltd. (Shanghai, China). These siRNAs were transfected into cells according to the manufacturer’s instructions.

### RT–qPCR

Total RNA was isolated using TRIzol® Reagent (Invitrogen) according to the manufacturer’s instructions. cDNA was reverse-transcribed using a reverse transcription kit (RR036A, TaKaRa, Tokyo, Japan). Real-time PCR was carried out using a SYBR Green PCR Kit (TaKaRa) and the Roche LightCycler 480 real-time PCR system [18]. The primers used were as follows: human *CCNG2* (Forward, 5′-TGC CTA GCC GAG TAT TCT TCT-3′; Reverse, 5′-TGT TTG TGC CAC TTT GAA GTT G-3′); human *CXCL9* (Forward, 5′-CTG TTC CTG CAT CAG CAC CAA C-3′; Reverse, 5′-TGA ACT CCA TTC TTC AGT GTA GCA-3′); human *IL-1β* (Forward, 5′-CCA CAG ACC TTC CAG GAG AAT G-3′; Reverse, 5′-GTG CAG TTC AGT GAT CGT ACA GG-3′); human *Arg-1* (Forward, 5′-TCA TCT GGG TGG ATG CTC ACA C-3′; Reverse, 5′-GAG AAT CCT GGC ACA TCG GGA A-3′); human *β-actin* (Forward, 5′-ATT GGC AAT GAG CGG TTC CG-3′; Reverse, 5′-CGT GGA TGC CAC AGG ACT CC-3′); mouse *CXCL9* (Forward, 5′-CCT AGT GAT AAG GAA TGC ACG ATG-3′; Reverse, 5′-CTA GGC AGG TTT GAT CTC CGT TC-3′); and mouse *GAPDH* (Forward, 5′-CAT CAC TGC CAC CCA GAA GAC TG-3′; Reverse, 5′-ATG CCA GTG AGC TTC CCG TTC AG-3′). Human *β-actin* and mouse *GAPDH* were used as internal controls.

### Western blotting and immunoprecipitation

Cells were lysed with RIPA buffer containing protease and phosphatase inhibitor cocktails (Roche, Basel, Switzerland), as previously described [16]. Equal amounts of protein were separated by SDS–PAGE and transferred to membranes. For immunoprecipitation, cell lysates were incubated with antibodies and Protein A/G Magnetic Beads (HY-K0202, MedChem Express), followed by western blotting. Western blotting was performed as previously described [19] with specific primary antibodies and horseradish peroxidase-conjugated secondary antibodies, followed by visualization using chemiluminescence (DNR Bio-Imaging Systems, Jerusalem, Israel). The antibodies used were as follows: anti-cyclin G2 (DF2284, Affinity Biosciences), anti-Flag (M20008XS, Abmart), anti-PP2Ac (2038 T, Cell Signaling Technology), anti-STAT1 (14994S, Cell Signaling Technology), anti-p-STAT1 (Y701) (9167S, Cell Signaling Technology), anti-lamin B1 (sc-6216, Santa Cruz), anti-β-tubulin (M30109XS, Abmart), anti-GAPDH (M20006F, Abmart), and anti-IgG (3900S, Cell Signaling Technology).

### Human peripheral blood monocytes isolation, culture and identification

Anticoagulant whole blood was diluted 1:1 with PBS (containing 5% FBS), then it was slowly added to the centrifuge tube containing lymphocytes separation medium (LTS10771, TBD), centrifuged at 1000 g for 20 min. The removed PBMC layer was resuspended in PBS, centrifuged at 300 g for 5 min, and discarded the supernatant. The previous step was repeated. Cells were resuspended and cultured in RPMI 1640 supplemented with 10% FBS, 1% penicillin/streptomycin, and 20 ng/mL M-CSF (216-MC-025/CF, R&D Systems). Macrophages were incubated with APC anti-human CD68 antibody (333,810, Biolegend), identified by flow cytometry.

### Bone marrow-derived macrophage (BMDM) isolation, culture and identification

The femur and tibia were removed from eight-week-old C57BL/6 mice, and single-cell suspensions were prepared in Hank’s Balanced Salt Solution (PB180323, Procell) containing 5% FBS. Red blood cells were lysed using red blood cell lysis buffer. The samples were centrifuged, and the supernatant was discarded. The cells were resuspended and cultured in RPMI 1640 supplemented with 10% FBS, 1% penicillin/streptomycin, and 20 ng/mL M-CSF (CB34, Novoprotein) [20]. BMDMs were incubated with APC anti-mouse/human CD11b antibody (101,212, Biolegend) and FITC anti-mouse F4/80 antibody (123,108, Biolegend), identified by flow cytometry.

### CTL isolation and culture

The spleens of C57BL/6 mice were removed, homogenized in RPMI 1640 medium, and filtered through a sterile 70-μm cell strainer. CD8^+^ T cells were isolated using the EasySep™ Mouse CD8^+^ T-Cell Isolation Kit (STEMCELL), according to the manufacturer’s instructions. Cells were cultured in RPMI 1640 medium containing 10% FBS, 1% penicillin/streptomycin, 0.05 mM β-mercaptoethanol (Sigma–Aldrich), 1 mM sodium pyruvate (Sigma–Aldrich), 2 mM L-glutamine (Invitrogen, Carlsbad, CA, USA), 20 ng/mL recombinant IL-2 (R&D Systems, Minneapolis), and 2 μg/mL anti-CD28 (Invitrogen). The cells were seeded into 5 μg/mL plate-bound anti-CD3 vessels (Invitrogen), as previously described [19].

### Animal experiments

*Ccng2* ^*−/−*^ C57BL/6 mice were described previously. The TALEN-targeted *Ccng2* knockout mice (*Ccng2*^*−/−*^) of the C57BL/6 N genetic background were generated by Cyagen (Cyagen Biosciences, Guangzhou, China) [16, 19]. LLC or MC38 cells (1.5 × 10^6^) were mixed with 3 × 10^5^ BMDMs from wild-type (WT) or *Ccng2*^*−/−*^ C57BL/6 mice and subcutaneously inoculated into the right flank of eight-week-old C57BL/6 mice. Each mouse was intraperitoneally injected with 2.5 μg IFN-γ (C746, Novoprotein) on Days 6, 9, and 12 after tumor cell inoculation. Tumors were periodically measured with calipers. The mice were euthanized on Day 15, and tumor weights and volumes were measured. All animal experiments were performed in accordance with relevant regulatory standards and approved by the Animal Ethics Committee of China Medical University.

### Immunochemistry

Mouse tissues were fixed and paraffin-embedded. The expression of Ki-67 (652,402, Biolegend), CD31 (AF3628, R&D Systems) and CD8a (98,941, Cell Signaling Technology) was determined using immunohistochemical assays, as previously described [16]. The slides were mounted with coverslips and photographed under a microscope (Nikon 80i).

### Immunofluorescence staining

Cells were grown on coverslips and incubated with an anti-STAT1 antibody (14994S, Cell Signaling Technology). Embedded mouse tissue was incubated with an anti-CD8a antibody (14–0081-82, Invitrogen). Both followed by incubation with corresponding secondary antibodies for 60 min at 37 °C in the dark. Nuclei were counterstained with DAPI. Photographs were taken with an Olympus LEXT OLS4500 Confocal Laser Scanning Microscope [17].

### Mouse tumor cell isolation

The tumors were placed in precooled Hank’s Balanced Salt Solution containing 5% FBS and cut to 1 mm with scissors. 1 mg/ mL collagenase I (SCR103, Sigma–Aldrich) and 1 mg/ mL DNase I (10,104,159,001, Roche) were used to digest tumor tissues in 37 °C water bath until tissue block dissolved. The single-cell suspensions were collected through 70 μm filters. Red blood cells were removed by red blood cell lysis buffer. Cells were washed and resuspended in stain buffer (FBS).

### Flow cytometry analysis

Cells were pretreated with a phorbol 12-myristate 13-acetate (PMA)/ionomycin mixture (70-CS1001, MultiSciences) and BFA/monensin mixture (70-CS1002, MultiSciences) for 5 h. The cells were then incubated with PE-labeled anti-mouse CD8a antibody (E-AB-F1104D, Elabscience) for 30 min at 4 °C in the dark. Subsequently, the cell membranes were disrupted using the Fixation/Permeabilization Kit (554,714, BD). The permeabilized cells were incubated with antibody at 4 °C for 30 min in the dark. The antibodies used included a recombinant APC-labeled anti-human/mouse granzyme B (372,204, Biolegend) and APC-labeled anti-mouse perforin (S16009B, Biolegend) antibodies. APC-labeled rat IgG2a and κ isotype control (E-AB-F09832E, Elabscience) and APC-labeled mouse IgG1 and κ isotype control (E-AB-F09792E, Elabscience) were used as negative controls. All cells were analyzed by flow cytometry using a BD Accuri C6 Plus Flow Cytometer.

### CTL chemotactic assay

Cell chemotaxis assays were performed in 24-well plates using a 5-μm chamber (Corning, NY, USA). A total of 10 × 10^4^ cells/well were suspended in RPMI 1640 containing 0.2% FBS in the upper chamber, and the lower chamber was filled with the conditioned medium of BMDMs from C57BL/6 mice with or without recombinant mouse CXCL9 protein (492-MM-010/CF, R&D Systems). The cells were cultured for 12 h in a humidified incubator at 37 °C with 5% CO_2_, and cells that invaded the lower surface were stained with 5 nM eBioscience™ Calcein AM Viability Dye (Invitrogen, USA) for 15 min and detected by fluorescence microscopy.

### Matrigel HUVEC and SVEC4–10 cell tube formation assays

A 96-well plate was coated with Matrigel (356,230, BD) at 37 °C for 30 min. HUVECs or SVEC4–10 cells (6 × 10^4^) were cultured in 150 μL of conditioned medium with or without recombinant human CXCL9 protein (392-MG-010/CF, R&D Systems), recombinant mouse CXCL9 protein for 2–6 h [21]. The cells were stained with 5 nM eBioscience™ Calcein AM Viability Dye for 15 min and detected by fluorescence microscopy. Tube formation was assessed using AngioTool, including estimating the vessel percentage areas, the total number of junctions, and total vessel length.

### Measurement of CXCL9 levels

Cell culture supernatants were collected, and human and mouse CXCL9 levels in the supernatants were quantified using the human and mouse CXCL9 Enzyme-Linked Immunosorbent Assay (ELISA) Kits (E-EL-H6062 and E-EL-M0020c, Elabscience), respectively.

### Stimulation of macrophages

THP-1 cells were treated with 100 ng/mL PMA (P8139, Sigma–Aldrich) for 48 h. The macrophages were then stimulated with 100 ng/mL IFN-γ (SRP3058, Sigma–Aldrich), 20 ng/mL LPS (916,374, Sigma–Aldrich), 20 ng/mL IL-4 (SRP3093, Sigma–Aldrich), or 20 ng/mL IL-13 (SRP3274, Sigma–Aldrich) for 48 h.

### Cytoplasmic and nuclear protein fractionation

Cytoplasmic and nuclear proteins were separated using a nuclear and cytoplasmic protein extraction kit (P0027, Beyotime), according to the manufacturer’s instructions.

### Statistical analysis

The means of the two groups were compared using the unpaired Student’s t-test. Data are presented as the mean ± SD. The weight and volume of tumors are presented as the mean ± SEM. The growth curves of the tumor volume were analyzed using two-way ANOVA. *p < 0.05; **p < 0.01; ***p < 0.001; ****p < 0.0001; ns, not significant.

## Results

### IFN-γ upregulates cyclin G2 expression in macrophages and inhibits lung cancer via cyclin G2

**Fig. 1:**
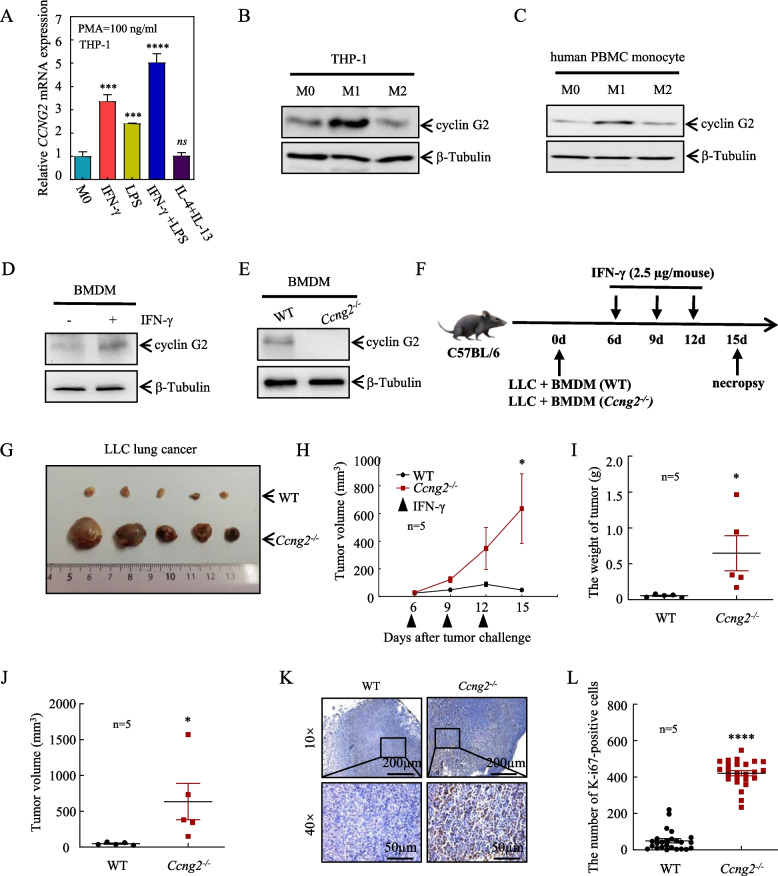
Knockout of cyclin G2 in macrophages attenuates the tumor suppressive effects of IFN-γ. **A** *CCNG2* mRNA expression in THP-1 cells treated with IFN-γ, LPS, IL-4 and IL-13 was detected RT–qPCR using *β-actin* as an internal control. Data are presented as the mean ± SD (Data of *CCNG2* mRNA expression in THP-1 cells treated with IFN-γ representing 3 independent experiments). **B** Cyclin G2 protein levels in THP-1 cells treated with IFN-γ, IL-4 and IL-13 were evaluated by western blotting using β-tubulin as a loading control (representing 3 independent experiments). **C** Cyclin G2 protein levels in the human peripheral blood monocytes were determined by western blotting following IFN-γ, IL-4 and IL-13 treatment. β-tubulin was used as a loading control (representing 3 independent experiments). **D** After IFN-γ (100 ng/mL) treatment of BMDMs, the cyclin G2 protein expression was detected by western blotting. β-tubulin was used as a loading control (representing 3 independent experiments). **E** BMDMs were isolated from WT and *Ccng2*^*−/−*^ C57BL/6 mice and identified by western blotting. β-tubulin was used as a loading control. **F** LLC cells were mixed with BMDMs from WT and *Ccng2*^*−/−*^ C57BL/6 mice at a ratio of 5:1 and injected subcutaneously into C57BL/6 mice, which were then treated with IFN-γ at specific times. **G–J** Gross tumors (G), tumor growth curves (H), and tumor weights (I) and volumes (J) measured at the study endpoint. (*n* = 5). **K** Representative Ki-67 immunohistochemical staining of tumors from mice in the WT and *Ccng2*^*−/−*^ groups. 10× scale bar = 200 μm; 40× scale bar = 50 μm. (L) Graph showing the number of Ki-67-positive cells in each field (*n* = 5). The data in H, I, J, and L are presented as the mean ± SEM. Data presented in A, I, J, and L were analyzed with the unpaired Student’s t-test. Data in H were analyzed using two-way ANOVA. *p < 0.05; ***p < 0.001; ****p < 0.0001; *ns*, not significant

To evaluate cyclin G2 expression in macrophages, THP-1 cells were differentiated into M1 or M2 macrophages by treating the cells with PMA and either IFN-γ or LPS or IL-4 + IL-13, respectively [22]. The macrophage types were confirmed by the expression of IL-1β (M1 marker) or Arg-1 (M2 marker) (Fig. S1A, S1B), and the *CCNG2* mRNA levels were measured in the two types of lineages. Compared to M0 macrophages, IFN-γ or LPS treatment alone or in combination upregulated *CCNG2* mRNA expression, with IFN-γ inducing greater levels. In contrast, the combined treatment with IL-4 and IL-13 did not significantly alter *CCNG2* mRNA expression (Fig. 1A). These changes were also observed at the cyclin G2 protein level in THP-1 cells (Fig. 1B). Similar results were also verified in human peripheral blood monocytes (Fig. 1C), cells were identified by the expression of CD68 (Fig. S1C). We also demonstrated that IFN-γ could upregulate cyclin G2 protein expression in BMDMs isolated from C57BL/6 mice (Fig. 1D), BMDMs were identified by the expression of CD11b and F4/80 (Fig. S1D).

Macrophages are the first line of communication between tumors and the rest of the immune system and play an extremely important role in tumor development [23]. Because M1 macrophages suppress tumors [24], we hypothesized that cyclin G2 might have an antitumor function in this macrophage type. Therefore, we isolated BMDMs from WT and *Ccng2*^−/−^ C57BL/6 mice (Fig. 1E), mixed them with LLC cells, and injected them subcutaneously into C57BL/6 mice, which were then treated with IFN-γ (Fig. 1F). The tumor growth rate in the *Ccng2*^−/−^ group was notably faster than the WT group after IFN-γ treatment, and the final tumor volumes and weights were larger (Fig. 1G–J). Moreover, there was an increase in the staining for the proliferation marker Ki-67 in the tumor tissues from the *Ccng2*^−/−^ group compared to the WT group (Fig. 1K, L). These results showed that *Ccng2* knockout in macrophages could attenuate the tumor suppressive effects of IFN-γ. Thus, our data demonstrated that M1 macrophages could upregulate cyclin G2 protein and suggested that cyclin G2 could promote the antitumor activity of M1 macrophages.

### Loss of cyclin G2 in macrophages reduces CTL recruitment, attenuating the tumor suppressive effects of IFN-γ

**Fig. 2:**
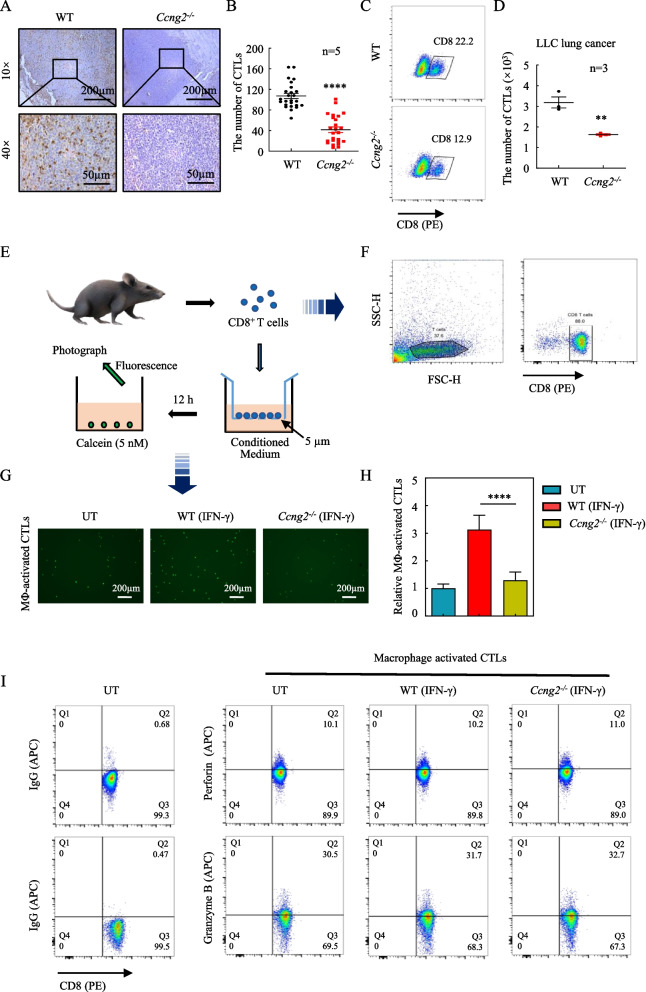
Depletion of cyclin G2 in macrophages reduces the chemotactic capacity of CTLs after IFN-γ treatment. **A** Representative CD8a immunohistochemistry staining of LLC tumors isolated from mice in the WT and *Ccng2*^*−/−*^ groups. 10× scale bar = 200 μm; 40× scale bar = 50 μm. **B** Graph of the number of CD8-positive cells in each field (*n* = 5). Data are presented as the mean ± SEM and were analyzed using the unpaired Student’s t-test. **C** The number of CD8^+^T cells of LLC tumors isolated from mice in the WT and *Ccng2*^*−/−*^ groups was evaluated by flow cytometry. **D** Data are presented as the mean ± SEM and were analyzed using the unpaired Student’s t-test (*n* = 3). **E** Schematic representation of the CTL chemotaxis assay. **F** Flow cytometric identification diagram for the CTLs. **G, H** Chemotaxis assays for CTLs treated with conditioned medium from BMDMs isolated from WT and *Ccng2*^*−/−*^ C57BL/6 mice. Scale bar = 200 μm. The data were compared to the untreated (UT) groups using the unpaired Student’s t-test. Data are presented as the mean ± SD (representing 3 independent experiments). **I** The expression levels of granzyme B and perforin in the CTLs after treatment with conditioned medium from BMDMs isolated from WT and *Ccng2*^*−/−*^ C57BL/6 mice were evaluated by flow cytometry. **p < 0.01; ****p < 0.0001

IFN-γ stimulates macrophages to secrete cytokines, which recruit CTLs [25, 26]. These CTLs recognize and eliminate cancer cells, thereby limiting tumor growth and metastasis [27]. Thus, we stained the tumor tissue collected from mice in the WT and *Ccng2*^*−/−*^ groups for CD8a. We found that the number of CD8^+^ T cells in the tumors from the *Ccng2*^−/−^ group was clearly reduced compared to tumors from the WT group (Fig. 2A, B, S2A and S2B). Similarly, the single-cell suspensions of tumor tissues from mice in the WT and *Ccng2*^*−/−*^ groups were collected to evaluate CD8^+^ T cells by flow cytometry, the results were consistent with the previous immunostaining (Fig. 2C, D and S2C). We next performed CTL chemotaxis experiments using CD8^+^ T cells isolated from the spleens of C57BL/6 mice (Fig. 2E, F) and conditioned medium from IFN-γ-treated BMDMs isolated from WT and *Ccng2*^*−/−*^ C57BL/6 mice. The number of CTLs in the *Ccng2*^*−/−*^ group was significantly lower than in the WT group, indicating that chemotaxis by the CTLs was significantly reduced after *Ccng2* knockout (Fig. 2G, H). Thus, loss of cyclin G2 in macrophages could effectively reduce CTL recruitment after IFN-γ treatment.

The cytotoxic function of CTLs is a key indicator of antitumor immunity [28], and granzyme B and perforin are markers of CTL cytotoxicity [29]. Interestingly, there were no significant differences in granzyme B and perforin expression between the *Ccng2*^*−/−*^ and WT groups (Fig. 2I). Taken together, these results demonstrated that *Ccng2* knockout in macrophages could reduce CTL recruitment in response to IFN-γ without affecting CTL cytotoxic functions.

### Deletion of *Ccng2* from macrophages promotes tumor angiogenesis and attenuates the tumor suppressive effects of IFN-γ

**Fig. 3:**
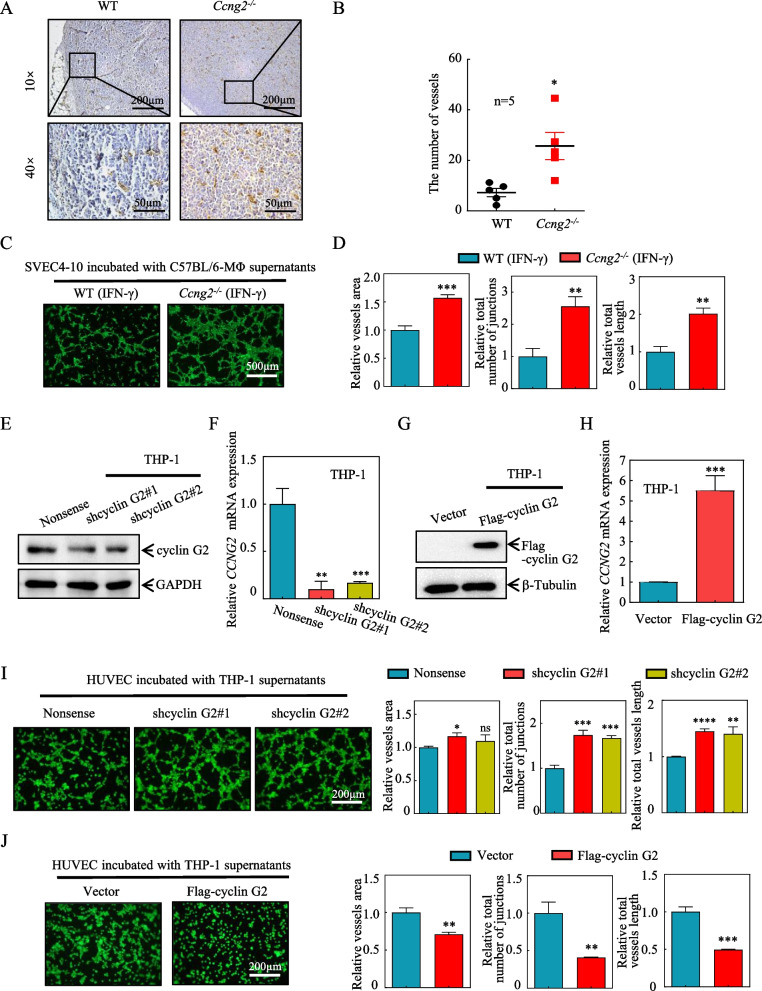
Cyclin G2 in macrophages suppresses tumors by inhibiting tumor blood vessels after IFN-γ treatment. **A** CD31 immunohistochemical staining of the LLC tumors isolated from mice in the WT and *Ccng2*^*−/−*^ groups, representative images are shown. 10× Scale bar = 200 μm; 40× Scale bar = 50 μm. **B** Graph showing the number of blood vessels in each field (*n* = 5). Data were analyzed using the unpaired Student’s t-test. Data are presented as the mean ± SEM. **C**,** D** Tube formation of SVEC4–10 cells treated with conditioned medium from BMDMs isolated from WT and *Ccng2*^*−/*−^ C57BL/6 mice. Scale bar = 500 μm (representing 3 independent experiments). **E** Western blot showing successful knockdown of cyclin G2 in THP-1 cells. GAPDH was used as a loading control. **F** RT-qPCR was used to measure *CCNG2* mRNA expression levels in THP-1 stable cell lines (Nonsense, shcyclin G2#1, and shcyclin G2#2). *β-actin* was used as an internal control. **G** Western blotting demonstrated successful cyclin G2 overexpression in THP-1 cells. β-tubulin was used as a loading control. **H** Measurement of *CCNG2* mRNA expression levels in THP-1 stable cell lines (Vector and Flag-cyclin G2) by RT-qPCR. *β-actin* was used as an internal control (representing 2 independent experiments). **I** Tube formation by HUVECs treated with conditioned medium from THP-1 stable cell lines (Nonsense, shcyclin G2#1, and shcyclin G2#2) cells. Scale bar = 200 μm (representing 3 independent experiments). **J** Tube formation by HUVECs treated with conditioned medium from THP-1 stable cell lines (Vector and Flag-cyclin G2) cells. Scale bar = 200 μm (representing 3 independent experiments). Data in D, F, H, I, and J were analyzed with the unpaired Student’s t-test. Data are presented as the mean ± SD. *p < 0.05; **p < 0.01; ***p < 0.001; ****p < 0.0001; *ns*, not significant

Macrophages act on vascular endothelial cells by secreting cytokines [30]. In the tumor microenvironment, abundant blood vessels provide sufficient nutrients for tumor cells and promote tumor progression [31]. Therefore, we examined mouse tumor tissues for the presence of blood vessels using the marker CD31. IHC revealed a greater density of microvessels in the tumors from the *Ccng2*^*−/−*^ group compared to the WT group (Fig. 3A, B). Thus, *Ccng2* knockout could promote angiogenesis. Moreover, we used conditioned medium from IFN-γ**-**treated BMDMs to determine its effect on the tube-forming ability of mouse vascular endothelial cells (SVEC4–10). The *Ccng2*^*−/−*^ conditioned medium promoted SVEC4–10 cell tube formation (Fig. 3C, D). To further evaluate this phenomenon, we generated stable shcyclin G2 (shcyclin G2#1 and shcyclin G2#2) and control (Nonsense), Vector and Flag-cyclin G2 THP-1 cell lines (Fig. 3E–H). Conditioned medium from these lines following treatment with IFN-γ was used for tube formation experiments with human umbilical vein endothelial cells (HUVECs). Cyclin G2 knockdown promoted HUVEC tube formation (Fig. 3I), whereas cyclin G2 overexpression inhibited this process (Fig. 3J). The above results demonstrated that cyclin G2 in both human and murine macrophages inhibited the tube formation of vascular endothelial cells after IFN-γ treatment.

### Loss of cyclin G2 reduces CTL recruitment and promotes vascular endothelial cell tube formation by decreasing CXCL9 secretion

**Fig. 4:**
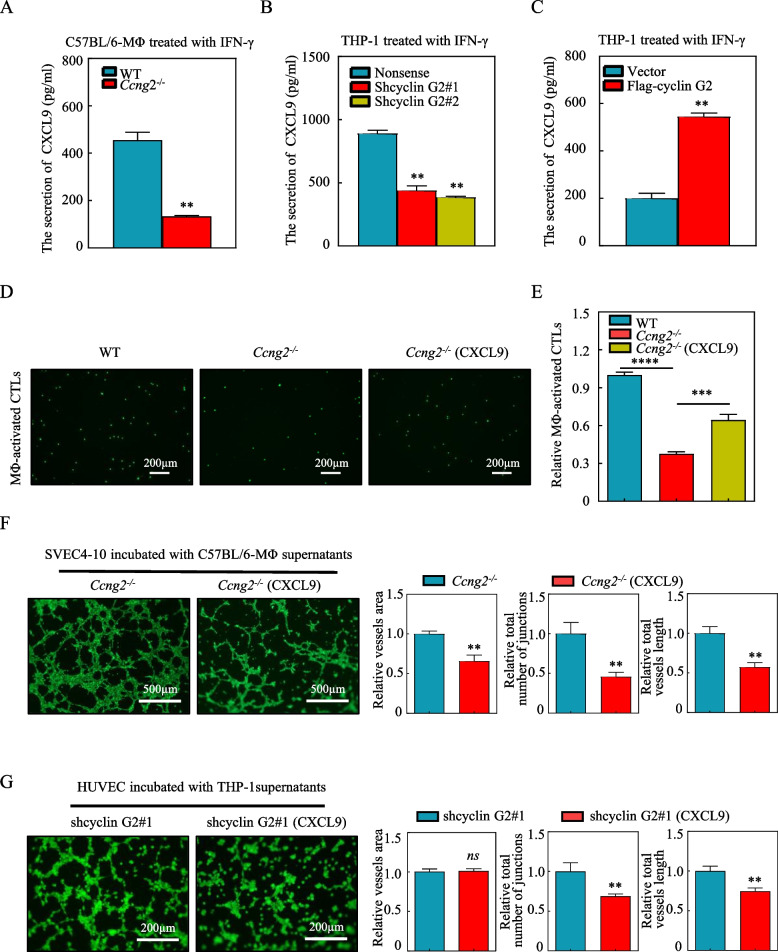
Cyclin G2 in macrophages regulates CTL chemotaxis and vascular endothelial cell tube formation via CXCL9. **A** CXCL9 levels in the supernatants of BMDMs from WT and *Ccng2*^*−/−*^ C57BL/6 mice treated with IFN-γ were determined by ELISA (representing 3 independent experiments). **B**,** C** CXCL9 levels in the supernatants of THP-1 stable cell lines (Nonsense, shcyclin G2#1, and shcyclin G2#2 and Vector and Flag-cyclin G2) treated with IFN-γ were determined by ELISA (representing 3 independent experiments). **D**,** E** CTL chemotaxis analyzed by treating conditioned medium from BMDMs isolated from WT and *Ccng2*^*−/−*^ C57BL/6 mice treated with or without recombinant CXCL9. Scale bar = 200 μm (representing 3 independent experiments). **F** Tube formation experiments showed the tube formation ability of SVEC4–10 cells treated with conditioned medium from BMDMs isolated from *Ccng2*^*−/−*^ C57BL/6 mice. The recombinant CXCL9 was added or not added to the conditioned medium. Scale bar = 500 μm (representing 3 independent experiments). **G** Tube formation experiments showed the tube formation ability of HUVECs treated with conditioned medium from a THP-1 stable cell line (shcyclin G2#1). The recombinant CXCL9 was added or not added to the conditioned medium. Scale bar = 200 μm (representing 3 independent experiments). (A–C, E–G) Data were analyzed with the unpaired Student’s t-test. Data are presented as the mean ± SD **p < 0.01; ***p < 0.001; *ns*, not significant

Next, we investigated what factor could be secreted by cyclin G2 knockout macrophages to regulate CTLs and vascular endothelial cells. It is known that the chemokine CXCL9 interacts with the CXCR3 receptor to modulate CTL recruitment in human cancers [32, 33]. Macrophages are the primary cells that secrete CXCL9, and CXCL9 production is significantly induced in an IFN-γ-dependent manner [32]. Therefore, we examined CXCL9 secretion from cyclin G2 knockout and WT macrophages after treatment with IFN-γ. We found that CXCL9 secretion from both *Ccng2*^*−/−*^ BMDMs and shcyclin G2 THP-1 cells decreased after IFN-γ treatment (Fig. 4A, B). Conversely, the conditioned medium from cyclin G2-overexpressing THP-1 cells increased CXCL9 secretion (Fig. 4C).

To verify that CXCL9 is the effector molecule, we added recombinant CXCL9 to the macrophage-conditioned medium and observed CTL chemotaxis. Indeed, the chemotactic capacity of the CTLs was restored after the addition of recombinant CXCL9 (Fig. 4D, E), confirming that knockout of cyclin G2 reduced the recruitment of CTLs by diminishing CXCL9 secretion. As CXCL9 has been reported to inhibit angiogenesis [10, 11], we further investigated whether CXCL9 was the downstream effector molecule for cyclin G2 involved in inhibiting angiogenesis. The addition of recombinant CXCL9 to the conditioned medium from macrophages inhibited tube formation by SVEC4–10 cells and HUVECs (Fig. 4F, G). These findings strongly suggested that reduced CXCL9 secretion attenuated the recruitment of CTLs and promoted vascular endothelial cell tube formation after cyclin G2 knockout.

### Cyclin G2 regulates CXCL9 transcription through the STAT1 signaling pathway

**Fig. 5:**
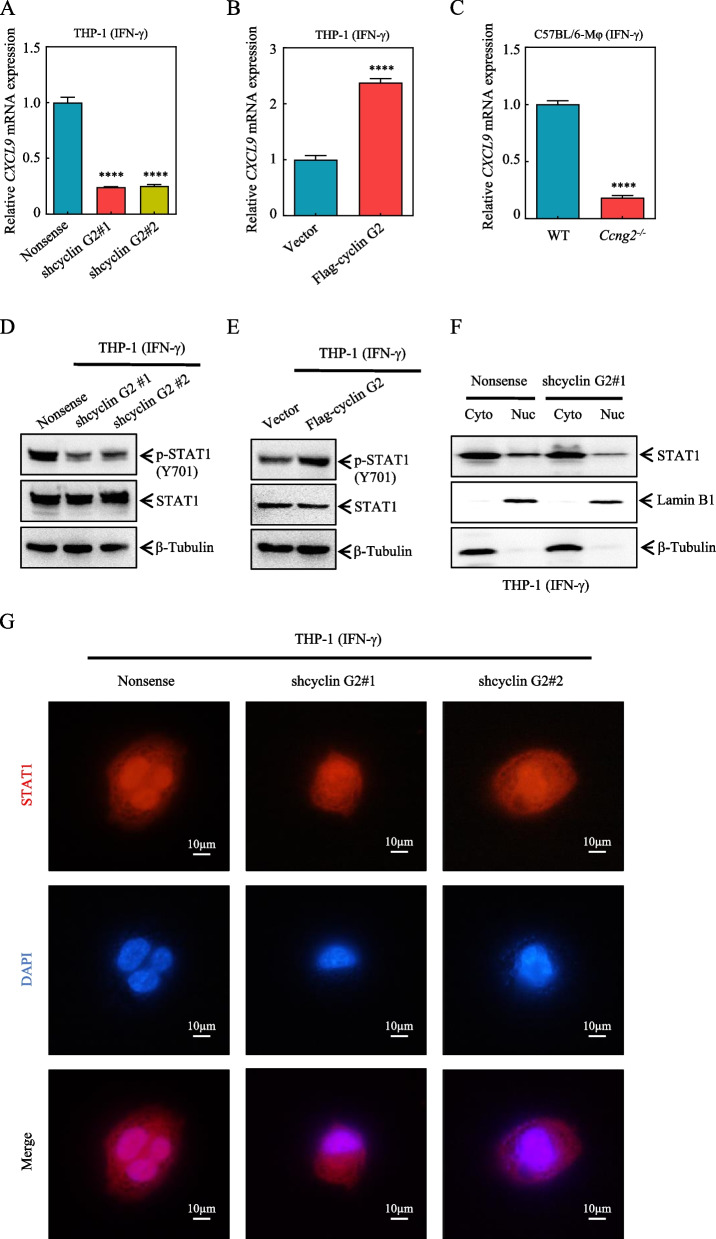
Cyclin G2 influences the activation of the STAT1-CXCL9 signaling pathway. **A**,** B** *CXCL9* mRNA levels in THP-1 stable cell lines (Nonsense, shcyclin G2#1, and shcyclin G2#2 and Vector and Flag-cyclin G2) as determined by RT–qPCR. *β-actin* was used as an internal control (representing 2 independent experiments). **C** Detection of *CXCL9* mRNA levels in BMDMs isolated from WT and *Ccng2*^*−/−*^ C57BL/6 mice by RT-qPCR. *GAPDH* was used as an internal control (representing 2 independent experiments). **D** The STAT1 and p-STAT1 (Y701) protein levels were determined in the THP-1 stable cell lines (Nonsense, shcyclin G2#1, and shcyclin G2#2) by western blotting. β-tubulin was used as a loading control. **E** The STAT1 and p-STAT1 (Y701) protein levels were determined in the THP-1 stable cell lines (Vector and Flag-cyclin G2) by western blotting. β-tubulin was used as a loading control (representing 3 independent experiments). **F** STAT1 protein levels in the cytoplasm and nucleus of THP-1 cells were detected by western blotting (Nonsense and shcyclin G2#1). β-tubulin was used as a cytoplasmic loading control. Lamin B1 was used as a nuclear loading control. **G** STAT1 immunofluorescence staining for THP-1 stable cell lines (Nonsense, shcyclin G2#1, and shcyclin G2#2), representative images are shown. Scale bar = 10 μm. (**A**– **C**) Data were analyzed with the unpaired Student’s t-test. Data are presented as the mean ± SD.****p < 0.0001

Based on the above results, we investigated the mechanism by which cyclin G2 regulates CXCL9 secretion in macrophages. First, we found that knockdown of cyclin G2 in macrophages decreased the *CXCL9* mRNA levels after IFN-γ treatment (Fig. 5A). Conversely, *CXCL9* mRNA levels increased after cyclin G2 overexpression (Fig. 5B). *CXCL9* mRNA expression was also downregulated in *Ccng2*^−/−^ BMDMs after IFN-γ treatment (Fig. 5C). These findings indicated that cyclin G2 could upregulate the *CXCL9* mRNA levels in macrophages.

IFN-γ can activate the phosphorylation of STAT1 via the JAK-STAT1 pathway, after which p-STAT1 (Y701) enters the nucleus and activates *CXCL9* gene transcription [34, 35]. Therefore, we evaluated STAT1 and p-STAT1 (Y701) protein levels in our experimental systems. After IFN-γ treatment, cyclin G2 knockdown decreased p-STAT1 (Y701) protein levels, and its overexpression had the opposite effect (Fig. 5D, E). Because STAT1 nuclear translocation is required for STAT1-mediated transcription [36], we examined STAT1 localization in macrophages. We observed reduced nuclear STAT1 levels after cyclin G2 knockdown (Fig. 5F, G). This finding indicated that cyclin G2 could promote STAT1 transport from the cytoplasm to the nucleus. Taken together, these observations demonstrated that cyclin G2 played a role in the activation of the STAT1 signaling pathway.

### STAT1 nuclear translocation mediated by cyclin G2 is dependent on PP2Ac in macrophages

**Fig. 6:**
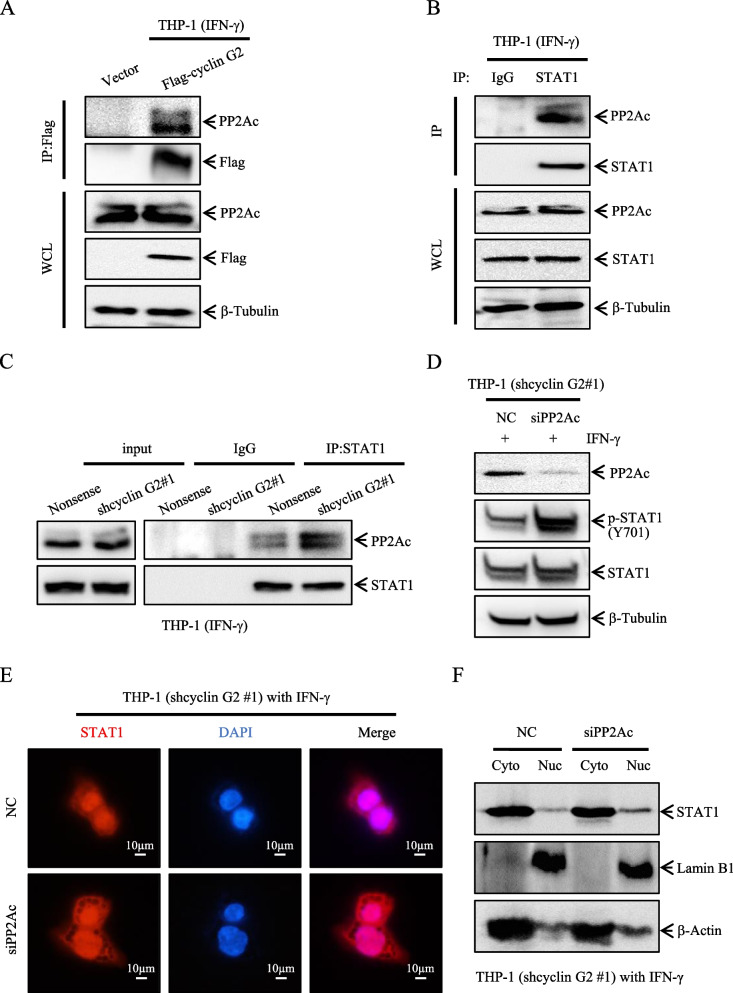
Cyclin G2 promotes STAT1 nuclear translocation via PP2Ac. **A** Western blotting of whole cell lysates (WCL) from THP-1 stable cell lines (Vector and Flag-cyclin G2) followed by IP with anti-Flag. β-Tubulin was used as a loading control. **B** Cell lysates from THP-1 cells were immunoprecipitated with STAT1 or IgG control antibody, followed by western blotting with a PP2Ac antibody. β-tubulin was used as a loading control. **C** Cell lysates from THP-1 stable cell lines (Nonsense and shcyclin G2#1) were immunoprecipitated with STAT1 or IgG control antibody, followed by western blot analysis with PP2Ac antibody. **D** STAT1 and p-STAT1 (Y701) protein levels were detected in THP-1 cell lines (shcyclin G2#1-NC and shcyclin G2#1-siPP2Ac) by western blotting. β-tubulin was used as a loading control. **E** siNC and siPP2Ac were transfected into THP-1 (shcyclin G2) cells respectively, and the nuclear content of STAT1 was detected by immunofluorescence, representative images were shown. Scale bar = 10 μm. **F** STAT1 protein expression in the cytoplasm and nucleus of THP-1 cell lines (shcyclin G2#1-NC and shcyclin G2#1-siPP2Ac) was detected by western blotting. β-actin was used as a cytoplasmic loading control. Lamin B1 was used as a nuclear loading control

Protein phosphatase 2 phosphatase activator (PP2A) was previously shown to attenuate IFN-γ-induced STAT1 phosphorylation [13–15]; cyclin G2 interacts with PP2Ac to affect its function [37–39]. Accordingly, we examined whether PP2Ac was involved in the change in STAT1 nuclear content in macrophages after cyclin G2 knockdown. IP revealed that both cyclin G2 and STAT1 interacted with PP2Ac in IFN-γ-stimulated THP-1 cells (Fig. 6A, B). Moreover, the amount of STAT1-bound PP2Ac was higher when cyclin G2 was knocked down (Fig. 6C). Therefore, cyclin G2 might compete with STAT1 for binding to PP2Ac in IFN-γ-stimulated THP-1 cells. Furthermore, we used siPP2Ac to knockdown the expression of PP2Ac in the shcyclin G2 THP-1 cells, the results revealed that knockdown of PP2Ac upregulated the levels of p-STAT1 (Y701) and nuclear STAT1 (Fig. 6D–F). Taken together, these results showed that IFN-γ-upregulated cyclin G2 increases the nuclear abundance of STAT1 in a PP2Ac-dependent manner.

### Cyclin G2 deficiency in macrophages attenuates the antitumor effect of IFN-γ in a colon cancer mouse model

**Fig. 7:**
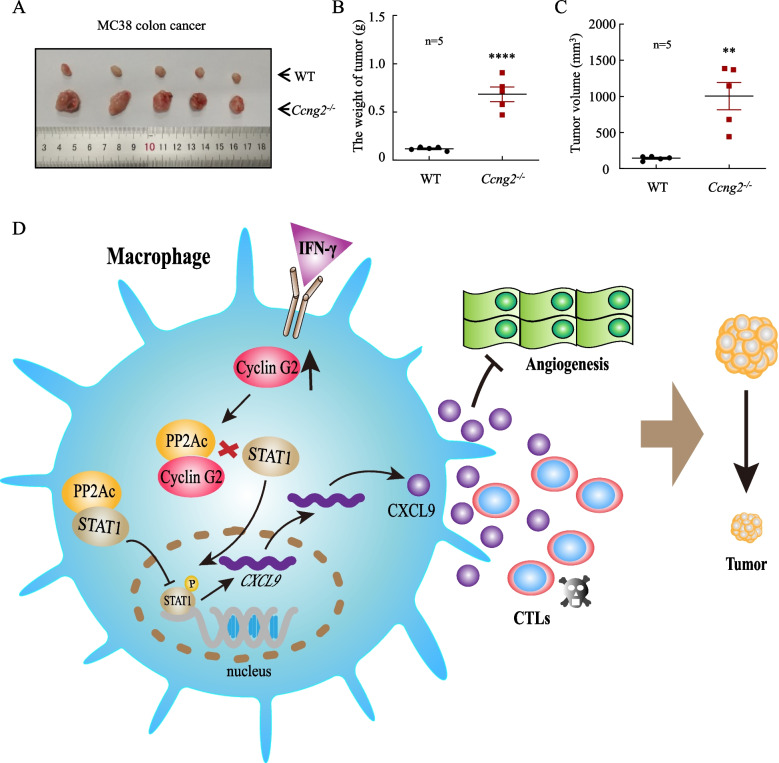
Cyclin G2 knockout in macrophages attenuates the inhibitory effects of IFN-γ on colon cancer cell growth. **A–C** MC38 cells were mixed with BMDMs from WT and *Ccng2*^*−/−*^ C57BL/6 mice at a ratio of 5:1 and injected subcutaneously into C57BL/6 mice, which were then treated with IFN-γ at specific times. Gross tumors (**A**), tumor weights (**B**), and tumor volumes (**C**) were measured at the endpoint. Data were analyzed with the unpaired Student’s t-test. Data are presented as the mean ± SEM (*n* = 5). **D** A schematic model depicting the role of cyclin G2 in macrophages after IFN-γ treatment. Upregulated cyclin G2 after IFN-γ treatment inhibited the interaction between PP2Ac and STAT1, thereby increasing the nuclear import of STAT1 and promoting *CXCL9* transcription. Increased CXCL9 secretion can promote CTL chemotaxis and inhibit vascular endothelial cell angiogenesis, ultimately inhibiting tumor progression. **p < 0.01; ****p < 0.0001

We previously found that cyclin G2 knockout in macrophages attenuated the antitumor effect of IFN-γ on lung cancer. To determine if loss of cyclin G2 had a similar effect on other cancers, we used colon cancer cells for further experiments. Namely, we mixed MC38 cells with BMDMs from WT or *Ccng2*^*−/−*^ C57BL/6 mice and subcutaneously injected them into C57BL/6 mice, then treated with IFN-γ. The final tumor volumes and weights were larger in the *Ccng2*^−/−^ group (Fig. 7A–C). Our results suggested that the function of cyclin G2 may be the same in many tumor types, including lung and colon cancer.

## Discussion

Preliminary studies by our group have shown that cyclin G2 acts as a tumor suppressor in various tumor cells [16–18]. However, its function in macrophages has not yet been explored. Here, we reveal that macrophage cyclin G2 alters the tumor microenvironment after IFN-γ treatment, suggesting that targeting cyclin G2 may be helpful for treating tumors.

IFN-γ is essential for innate, adaptive, and antitumor immunity [40, 41]. We found that it can upregulate cyclin G2 protein expression in macrophages, and cyclin G2 activates the IFN-γ-STAT1 signaling pathway, thereby affecting the tumor microenvironment. We next sought to determine whether cyclin G2 might also be involved in innate and adaptive immunity in response to bacterial and viral infections.

We found that subcutaneous lung and colon tumors grew larger after cyclin G2 deletion from macrophages after IFN-γ treatment, indicating that cyclin G2 in macrophages alters the tumor microenvironment and plays a key role in tumor suppression mediated by IFN-γ-stimulated macrophages. Although we only explored the role of cyclin G2 in lung and colon cancers, macrophages are ubiquitous in the tumor microenvironment of various cancers and have crucial functions. Therefore, we speculate that cyclin G2 knockout in macrophages may affect the progression of more cancer types.

CTLs are the main antitumor effector cells [42–44]. IFN-γ stimulates macrophages to recruit more CTLs to remodel the tumor microenvironment. In many cancer types, the presence of more CTLs around the tumor is considered a favorable prognostic indicator. We found that cyclin G2 plays a critical role in the recruitment of CTLs by IFN-γ-stimulated macrophages, thereby remodeling the tumor microenvironment. Moreover, tumor angiogenesis plays an important role in the occurrence and development of tumors; blocking tumor angiogenesis is a feasible means of anticancer therapy [45]. We also found that cyclin G2 could inhibit tumor angiogenesis after IFN-γ stimulation of macrophages. These data suggest that cyclin G2 in macrophages plays a crucial role in IFN-γ-mediated remodeling of the tumor microenvironment. Therefore, identifying treatments that can modify the tumor microenvironment through cyclin G2 is worth further study.

CXCL9 is an important effector of IFN-γ-induced macrophages. CXCL9 primarily plays a role in T-cell attraction and antiangiogenesis [46]. CTLs express CXCR3, the receptor for CXCL9, and migrate along a gradient of CXCL9 [47]. CXCL9 also acts on vascular endothelial cells to inhibit tumor angiogenesis [48]. IFN-γ plays an important role in regulating the tumor immune microenvironment by inducing the production of CXCL9. CXCL9 is upregulated in chemotherapy-sensitive patient tumors and increases T-cell infiltration, tumor control, and patient survival. Therefore, CXCL9 may represent a new strategy for improving the efficacy of cancer immunotherapy [49]. We found that cyclin G2 increased CXCL9 production and secretion from macrophages after IFN-γ treatment, which could explain the effects of cyclin G2 on CTL recruitment and tumor angiogenesis.

IFN-γ activates macrophages by promoting STAT1 signaling [50]. IFN-γ stimulates the phosphorylation, homodimerization, and nuclear translocation of STAT1, promoting *CXCL9* transcription [36]. Indeed, STAT1 nuclear translocation is an important step in IFN-γ signaling. PP2Ac is a negative regulator of the IFN pathway, and studies have shown that PP2Ac inhibits IFN-induced STAT1 phosphorylation, resulting in reduced nuclear STAT1 levels [14]. In our study, cyclin G2 promoted the translocation of STAT1 from the cytoplasm to the nucleus. Moreover, cyclin G2 could inhibit the interaction between PP2Ac and STAT1. However, the specific binding regions of cyclin G2 and STAT1 to PP2Ac need further investigation.

We found that IFN-γ up regulates the expression of cyclin G2, increased cyclin G2 in macrophages of the TME augments the antitumor effects of IFN-γ. In Fig. 3J, overexpression of cyclin G2 inhibits angiogenesis with IFN-γ treatment. Therefore, we speculate that overexpression of cyclin G2 in macrophages can enhance effect of IFN-γ treat tumor, which may be a potential therapeutic scheme. Our previous research found that cyclin G2 plays an anti-tumor role in a variety of cancer cells, and overexpression of cyclin G2 in glioma combined with PD-1 therapy can reverse the immunosuppressive tumor microenvironment. Therefore, we propose to inject lentiviruses or preparations (overexpressing cyclin G2) into the tumor of patients to make cancer cells and macrophages overexpress cyclin G2, then combine IFN-γ or IFN-γ + PD-1 inhibitor to suppress tumor. This may achieve a wonderful therapeutic effect.

In conclusion, we found that IFN-γ could upregulate cyclin G2 expression in macrophages. Cyclin G2 inhibited tumor angiogenesis and promoted the recruitment of CTLs by increasing the release of CXCL9 from macrophages. This process occurred when IFN-γ stimulated macrophages to remodel the tumor microenvironment. This study is the first report showing that cyclin G2 promotes STAT1 nuclear translocation by inhibiting the interaction between PP2Ac and STAT1, thus increasing CXCL9 production. Therefore, the tumor suppressive effects of cyclin G2 are mediated through its regulation of CXCL9 secretion from macrophages under the action of IFN-γ. In addition, macrophage cyclin G2 may be a biomarker for treatment sensitivity and prognosis during the selection of cancer patients for immunotherapy.

## Conclusions

Cyclin G2 is upregulated by IFN-γ in macrophages, promotes the secretion of CXCL9 to increase CTL chemotaxis and inhibit angiogenesis to suppress tumor growth. Our findings suggest that targeting cyclin G2 could benefit future immunotherapy.

## Supplementary Information

**Additional file 1: Fig. S1.** Identification of macrophages. (A) *IL-1β* mRNA expression in macrophages treated with IFN-γ or LPS was examined using RT–qPCR. *β-actin* was used as an internal control. (B) *Arg-1* mRNA expression in macrophages treated with IL-4 and IL-13 was evaluated by RT–qPCR. *β-actin* was used as an internal control. Data were analyzed using the unpaired Student’s t-test. Data are presented as the mean ± SD. (C) The expression level of CD68 in macrophages isolated from human peripheral blood was evaluated by flow cytometry. (D) The expression levels of CD11b and F4/80 in BMDMs isolated from C57BL/6 mice were examined by flow cytometry. **p < 0.01; ****p < 0.0001. **Fig. S2.** Immunofluorescence staining of CD8^+^ T cells. (A) Representative CD8a immunofluorescence staining of LLC tumors isolated from mice in the WT and *Ccng2*^*−/−*^ groups. Scale bar = 50 μm. (B) Graph of the number of CD8-positive cells in each field (*n* = 5). Data are presented as the mean ± SEM and were analyzed using the unpaired Student’s t-test. (C) Flow gate diagram of CD8^+^ T cells from LLC tumors. ****p < 0.0001.

## Data Availability

The datasets used and/or analyzed during the current study are available from the corresponding author upon reasonable request.

## Abbreviations

CTLs: Cytotoxic T lymphocytes
IFN-γ: Interferon-gamma
IP: Immunoprecipitation
ELISAs: Enzyme-linked immunosorbent assays
STAT1: Signal transducer and activator of transcription 1
BMDMs: Bone marrow-derived macrophages
PMA: Phorbol 12-myristate 13-acetate
PP2A: Protein phosphatase 2 phosphatase activator
