# Supplementary figures and images for: Cyclin G2 in macrophages triggers CTL-mediated antitumor immunity and antiangiogenesis via interferon-gamma
Source: J Exp Clin Cancer Res. 2022 Dec 24;41:358. doi: 10.1186/s13046-022-02564-2 (PMC9789679; doi:10.1186/s13046-022-02564-2)

Fig.S1

A

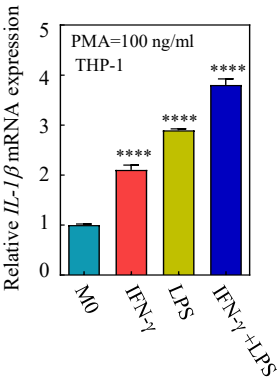

B

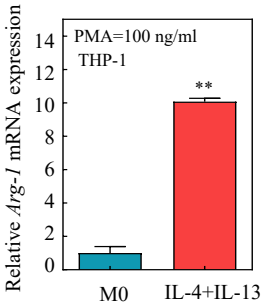

C

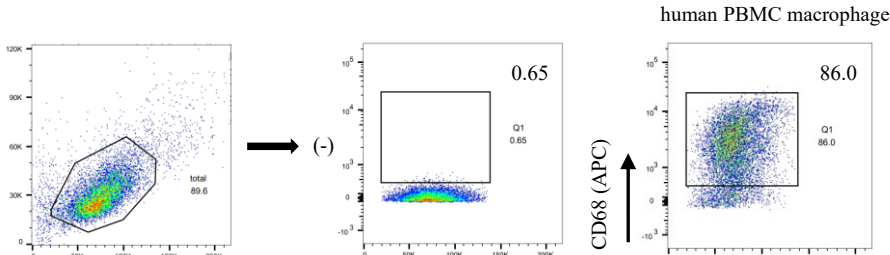

D

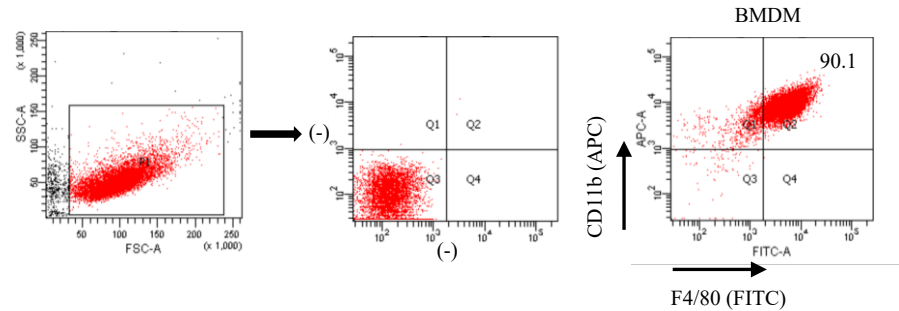

Fig.S2

A

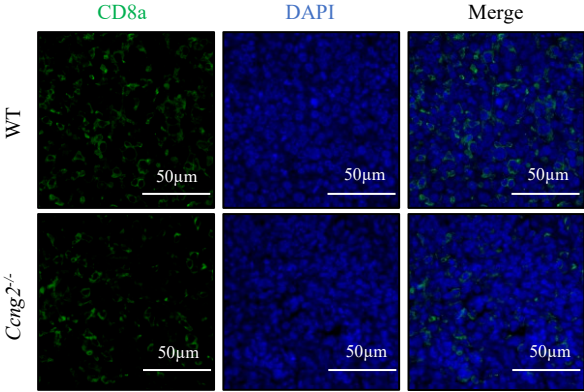

B

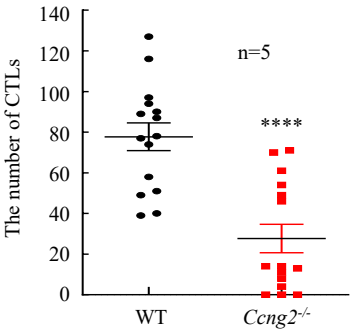

C

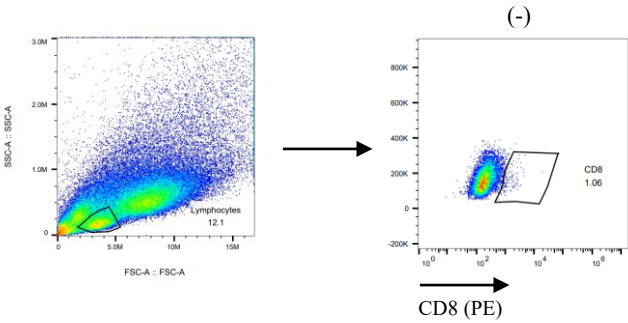

Supplement: Supplementary file 1 — Additional file 1: Fig. S1. Identification of macrophages. (A) IL-1β mRNA expression in macrophages treated with IFN-γ or LPS was examined using RT–qPCR. β-actin was used as an internal control. (B) Arg-1 mRNA expression in macrophages treated with IL-4 and IL-13 was evaluated by RT–qPCR. β-actin was used as an internal control. Data were analyzed using the unpaired Student’s t-test. Data are presented as the mean ± SD. (C) The expression level of CD68 in macrophages isolated from human peripheral blood was evaluated by flow cytometry. (D) The expression levels of CD11b and F4/80 in BMDMs isolated from C57BL/6 mice were examined by flow cytometry. **p < 0.01; ****p < 0.0001. Fig. S2. Immunofluorescence staining of CD8+ T cells. (A) Representative CD8a immunofluorescence staining of LLC tumors isolated from mice in the WT and Ccng2−/− groups. Scale bar = 50 μm. (B) Graph of the number of CD8-positive cells in each field (n = 5). Data are presented as the mean ± SEM and were analyzed using the unpaired Student’s t-test. (C) Flow gate diagram of CD8+ T cells from LLC tumors. ****p < 0.0001. [file 13046_2022_2564_MOESM1_ESM.pdf]
